# Longitudinal measurement and hierarchical classification framework for the prediction of Alzheimer’s disease

**DOI:** 10.1038/srep39880

**Published:** 2017-01-12

**Authors:** Meiyan Huang, Wei Yang, Qianjin Feng, Wufan Chen, The Alzheimer’s Disease Neuroimaging Initiative, Michael W. Weiner, Paul Aisen, Ronald Petersen, Clifford R. Jack, William Jagust, John Q. Trojanowki, Arthur W. Toga, Laurel Beckett, Robert C. Green, Andrew J. Saykin, John Morris, Leslie M. Shaw, Jeffrey Kaye, Joseph Quinn, Lisa Silbert, Betty Lind, Raina Carter, Sara Dolen, Lon S. Schneider, Sonia Pawluczyk, Mauricio Beccera, Liberty Teodoro, Bryan M. Spann, James Brewer, Helen Vanderswag, Adam Fleisher, Judith L. Heidebrink, Joanne L. Lord, Sara S. Mason, Colleen S. Albers, David Knopman, Kris Johnson, Rachelle S. Doody, Javier Villanueva-Meyer, Munir Chowdhury, Susan Rountree, Mimi Dang, Yaakov Stern, Lawrence S. Honig, Karen L. Bell, Beau Ances, John C. Morris, Maria Carroll, Mary L. Creech, Erin Franklin, Mark A. Mintun, Stacy Schneider, Angela Oliver, Daniel Marson, Randall Griffith, David Clark, David Geldmacher, John Brockington, Erik Roberson, Marissa Natelson Love, Hillel Grossman, Effie Mitsis, Raj C. Shah, Leyla deToledo-Morrell, Ranjan Duara, Daniel Varon, Maria T. Greig, Peggy Roberts, Marilyn Albert, Chiadi Onyike, Daniel D’Agostino, Stephanie Kielb, James E. Galvin, Brittany Cerbone, Christina A. Michel, Dana M. Pogorelec, Henry Rusinek, Mony J. de Leon, Lidia Glodzik, Susan De Santi, P. Murali Doraiswamy, Jeffrey R. Petrella, Salvador Borges-Neto, Terence Z. Wong, Edward Coleman, Charles D. Smith, Greg Jicha, Peter Hardy, Partha Sinha, Elizabeth Oates, Gary Conrad, Anton P. Porsteinsson, Bonnie S. Goldstein, Kim Martin, Kelly M. Makino, M. Saleem Ismail, Connie Brand, Ruth A. Mulnard, Gaby Thai, Catherine Mc-Adams-Ortiz, Kyle Womack, Dana Mathews, Mary Quiceno, Allan I. Levey, James J. Lah, Janet S. Cellar, Jeffrey M. Burns, Russell H. Swerdlow, William M. Brooks, Liana Apostolova, Kathleen Tingus, Ellen Woo, Daniel H. S. Silverman, Po H. Lu, George Bartzokis, Neill R. Graff-Radford, Francine Parfitt, Tracy Kendall, Heather Johnson, Martin R. Farlow, Ann Marie Hake, Brandy R. Matthews, Jared R. Brosch, Scott Herring, Cynthia Hunt, Christopher H. van Dyck, Richard E. Carson, Martha G. MacAvoy, Pradeep Varma, Howard Chertkow, Howard Bergman, Chris Hosein, Sandra Black, Bojana Stefanovic, Curtis Caldwell, Ging-Yuek Robin Hsiung, Howard Feldman, Benita Mudge, Michele Assaly, Elizabeth Finger, Stephen Pasternack, Irina Rachisky, Dick Trost, Andrew Kertesz, Charles Bernick, Donna Munic, Marek Marsel Mesulam, Kristine Lipowski, Sandra Weintraub, Borna Bonakdarpour, Diana Kerwin, Chuang-Kuo Wu, Nancy Johnson, Carl Sadowsky, Teresa Villena, Raymond Scott Turner, Kathleen Johnson, Brigid Reynolds, Reisa A. Sperling, Keith A. Johnson, Gad Marshall, Jerome Yesavage, Joy L. Taylor, Barton Lane, Allyson Rosen, Jared Tinklenberg, Marwan N. Sabbagh, Christine M. Belden, Sandra A. Jacobson, Sherye A. Sirrel, Neil Kowall, Ronald Killiany, Andrew E. Budson, Alexander Norbash, Patricia Lynn Johnson, Thomas O. Obisesan, Saba Wolday, Joanne Allard, Alan Lerner, Paula Ogrocki, Curtis Tatsuoka, Parianne Fatica, Evan Fletcher, Pauline Maillard, John Olichney, Charles DeCarli, Owen Carmichael, Smita Kittur, Michael Borrie, T-Y Lee, Rob Bartha, Sterling Johnson, Sanjay Asthana, Cynthia M. Carlsson, Steven G. Potkin, Adrian Preda, Dana Nguyen, Pierre Tariot, Anna Burke, Nadira Trncic, Adam Fleisher, Stephanie Reeder, Vernice Bates, Horacio Capote, Michelle Rainka, Douglas W. Scharre, Maria Kataki, Anahita Adeli, Earl A. Zimmerman, Dzintra Celmins, Alice D. Brown, Godfrey D. Pearlson, Karen Blank, Karen Anderson, Laura A. Flashman, Marc Seltzer, Mary L. Hynes, Robert B. Santulli, Kaycee M. Sink, Leslie Gordineer, Jeff D. Williamson, Pradeep Garg, Franklin Watkins, Brian R. Ott, Henry Querfurth, Geoffrey Tremont, Stephen Salloway, Paul Malloy, Stephen Correia, Howard J. Rosen, Bruce L. Miller, David Perry, Jacobo Mintzer, Kenneth Spicer, David Bachman, Nunzio Pomara, Raymundo Hernando, Antero Sarrael, Norman Relkin, Gloria Chaing, Michael Lin, Lisa Ravdin, Amanda Smith, Balebail Ashok Raj, Kristin Fargher

**Affiliations:** 1 Guangdong Provincial Key Laboratory of Medical Image Processing, School of Biomedical Engineering, Southern Medical University, Guangzhou, China; 2 Magnetic Resonance Unit at the VA Medical Center and Radiology, Medicine, Psychiatry and Neurology, University of California, San Francisco, USA; 3 San Diego School of Medicine, University of California, California, USA; 4 Mayo Clinic, Minnesota, USA; 5 Mayo Clinic, Rochester, USA; 6 University of California, Berkeley, USA; 7 University of Pennsylvania, Pennsylvania, USA; 8 University of Southern California, California, USA; 9 University of California, Davis, California, USA; 10 MPH Brigham and Women’s Hospital/Harvard Medical School, Massachusetts, USA; 11 Indiana University, Indiana, USA; 12 Washington University St. Louis, Missouri, USA; 13 Oregon Health and Science University, Oregon, USA; 14 University of California–San Diego, California, USA; 15 University of Michigan, Michigan, USA; 16 Baylor College of Medicine, Houston, State of Texas, USA; 17 Columbia University Medical Center, South Carolina, USA; 18 University of Alabama–Birmingham, Alabama, USA; 19 Mount Sinai School of Medicine, New York, USA; 20 Rush University Medical Center, Rush University, Illinois, USA; 21 Wien Center, Florida, USA; 22 Johns Hopkins University, Maryland, USA; 23 New York University, NY, USA; 24 Duke University Medical Center, North Carolina, USA; 25 University of Kentucky, Kentucky, USA; 26 University of Rochester Medical Center, NY, USA; 27 University of California, Irvine, California, USA; 28 University of Texas Southwestern Medical School, Texas, USA; 29 Emory University, Georgia, USA; 30 University of Kansas, Medical Center, Kansas, USA; 31 University of California, Los Angeles, California, USA; 32 Mayo Clinic, Jacksonville, USA; 33 Yale University School of Medicine, Connecticut, USA; 34 McGill University, Montreal-Jewish General Hospital, Montreal, Quebec, Canada; 35 Sunnybrook Health Sciences, Ontario, USA; 36 U.B.C. Clinic for AD & Related Disorders, Vancouver, Canada; 37 Cognitive Neurology - St. Joseph’s, Ontario, USA; 38 Cleveland Clinic Lou Ruvo Center for Brain Health, Ohio, USA; 39 Northwestern University, Illinois, USA; 40 Premiere Research Inst (Palm Beach Neurology), Florida, USA; 41 Georgetown University Medical Center, Washington D.C, USA; 42 Brigham and Women’s Hospital, Massachusetts, USA; 43 Stanford University, California, USA; 44 Banner Sun Health Research Institute, Arizona, USA; 45 Boston University, Massachusetts, USA; 46 Howard University, Washington D.C, USA; 47 Case Western Reserve University, Ohio, USA; 48 University of California, Davis–Sacramento, California, USA; 49 Neurological Care of CNY, New York, USA; 50 Parkwood Hospital, Pennsylvania, USA; 51 University of Wisconsin, Wisconsin, USA; 52 University of California, Irvine – BIC, California, USA; 53 Banner Alzheimer’s Institute, Arizona, USA; 54 Dent Neurologic Institute, NY, USA; 55 Ohio State University, Ohio, USA; 56 Albany Medical College, NY, USA; 57 Hartford Hospital, Olin Neuropsychiatry Research Center, Connecticut, USA; 58 Dartmouth-Hitchcock Medical Center, New Hampshire, USA; 59 Wake Forest University Health Sciences, North Carolina, USA; 60 Rhode Island Hospital, state of Rhode Island, USA; 61 Butler Hospital, Providence, Rhode Island, USA; 62 University of California, San Francisco, USA; 63 Medical University South Carolina, South Carolina, USA; 64 Nathan Kline Institute, Orangeburg, New York, USA; 65 Cornell University, Ithaca, New York, USA; 66 USF Health Byrd Alzheimer’s Institute, University of South Florida, Florida, USA

## Abstract

Accurate prediction of Alzheimer’s disease (AD) is important for the early diagnosis and treatment of this condition. Mild cognitive impairment (MCI) is an early stage of AD. Therefore, patients with MCI who are at high risk of fully developing AD should be identified to accurately predict AD. However, the relationship between brain images and AD is difficult to construct because of the complex characteristics of neuroimaging data. To address this problem, we present a longitudinal measurement of MCI brain images and a hierarchical classification method for AD prediction. Longitudinal images obtained from individuals with MCI were investigated to acquire important information on the longitudinal changes, which can be used to classify MCI subjects as either MCI conversion (MCIc) or MCI non-conversion (MCInc) individuals. Moreover, a hierarchical framework was introduced to the classifier to manage high feature dimensionality issues and incorporate spatial information for improving the prediction accuracy. The proposed method was evaluated using 131 patients with MCI (70 MCIc and 61 MCInc) based on MRI scans taken at different time points. Results showed that the proposed method achieved 79.4% accuracy for the classification of MCIc versus MCInc, thereby demonstrating very promising performance for AD prediction.

Alzheimer’s disease (AD) is characterized by the progressive impairment of cognitive and memory functions and is the most common form of dementia in elderly people. As the life expectancy increases, the number of AD patients increases accordingly, thereby causing a heavy socioeconomic burden^1^,^2^. Mild cognitive impairment (MCI) is a prodromal stage of AD; existing studies have suggested that individuals with amnestic MCI tend to progress to probable AD at a rate of approximately 10% to 15% per year^1^,^3^. Generally, patients with MCI who convert to AD after some time are called MCI converters (MCIc), whereas others who never convert to AD or even revert to a normal status are called MCI non-converters (MCInc). The classification of MCInc and MCIc is studied because of its importance in the early prediction of AD. Considering the limited period for which the symptomatic treatments are effective, patients with MCI who are at high risk of fully developing AD should be identified.

Recent studies showed that MRI can contribute significant progress to understand the neural changes related to AD and other diseases. Moreover, MRI data provide some brain structure information; this information can be used to identify the anatomical differences between populations of AD patients and normal controls (NC) and assist in the diagnosis and evaluation of MCI progression^4^,^5^. Generally, most MRI-based classification methods consist of two major steps: (1) feature extraction and selection and (2) classifier learning. Basing on the type of features extracted from MRI, the MCInc/MCIc classification methods can be divided into three categories: the voxel-based approach^6^,^7^,^8^,^9^, the vertex-based approach^1^,^10^,^11^, and the region of interest (ROI)-based approach^12^,^13^,^14^,^15^.

The vertex-based approach can be used to obtain information regarding the conversion from MCI to AD by using cortical thickness, sulcal depth, or cortical surface area as features. Although crucial disease progression information can be acquired through the vertex-based approach, this method depends on the accuracy of the surface registration^1^. The ROI-based approach usually employs nonlinear registration to register a brain MRI image to a structurally or functionally predefined brain region template before extracting representative features from each region. Although the ROI-based approach can significantly reduce the feature dimensionality, the features extracted from ROIs are very coarse and cannot reflect small or subtle changes associated with the brain diseases^16^. In the voxel-based approach, the features are defined at the level of the MRI voxel, which is simple and intuitive in terms of the interpretation of the results. However, the main limitations of the voxel-based approach are the high dimensionality of feature vectors and the lack of spatial information^16^. On one hand, the high dimensionality of feature vectors often leads to low performance attributed to the “curse of dimensionality”^2^. To address this problem, feature selection is typically performed to reduce feature dimensionality and eliminate the redundant features. On the other hand, AD often affects spatially contiguous regions instead of isolated voxels. Thus, the local spatial contiguity of the selected discriminative features (voxels) should be carefully considered during feature selection or classification^2^,^5^,^16^.

Recently, several longitudinal neuroimaging studies have collected a rich set of longitudinal data to better understand the progress of neuropsychiatric and neurodegenerative diseases or normal brain development^3^,^17^,^18^,^19^,^20^,^21^,^22^. The predictive value of early brain developmental trajectories should be studied for later brain and cognitive development and disease progression^18^,^19^. Therefore, the longitudinal changes in MRI measures may be a crucial factor in the prediction of future conversion from MCI to AD^3^,^7^,^20^,^23^,^24^,^25^. In the group-based approaches, longitudinal data have already been used for measuring longitudinal changes of the brain; until very recently, only a few researchers started to use longitudinal data for individual-based MCInc/MCIc classification^3^,^7^,^20^,^26^,^27^. Li *et al*.^20^ investigated the longitudinal cortical thickness changes of 75 MCI subjects to distinguish MCIc from MCInc. Moreover, Zhang *et al*.^3^ proposed an AD prediction method with ROI-based features from longitudinal data. The experiments were performed on 88 MCI subjects, and the results shown that the performance of their method with longitudinal data was better than that with baseline visit data^3^. Despite these efforts, extracting discriminative features from longitudinal data for the early diagnosis and prediction of AD progression is still challenging and requires more research.

In the present study, a longitudinal measurement-based hierarchical classification (LMHC) method for AD prediction is proposed. Specifically, longitudinal images obtained from individuals with MCI were investigated to acquire important information on the longitudinal changes that can be used to classify MCI subjects as MCIc or MCInc individuals. From a clinical perspective, an observed trend can show the tendency of an MCI subject to become an AD patient or to remain stable. If such trends are dynamically monitored with longitudinal data, AD-related changes can be determined; an AD prediction model can be constructed with the longitudinal data. In clinical settings, when a new MRI scan of an MCI subject is available, the future medical condition of the MCI subject (to develop AD or remain stable) can be predicted with his/her previous MRI scans and the constructed prediction model (Fig. 1). Thus, richer information can be extracted from the longitudinal data to help enhance the prediction accuracy. From a feature extraction perspective, several studies also suggested that voxel-based morphometry of longitudinal data can provide useful information regarding AD progression^25^,^28^,^29^. Thus, voxel intensities in MRI images from longitudinal data are used as features for classification. From a classifier construction perspective, instead of building a single classifier with an optimal subset of features, an ensemble learning method was used in this study to improve the generalizability and robustness of individual classifiers. Recently, a hierarchical ensemble classification method that combined multilevel classifiers through gradual integration of numerous features from both local brain regions and interbrain regions was proposed in refs 16 and 30. Unlike the abovementioned studies, we proposes a hierarchical classification method that builds multiple and multilevel classifiers with supervised learning and suitable thresholds to address the issues of high feature dimensionality and sensitivity to small changes for more accurate classification of MCI. Therefore, we can evaluate the classification abilities of the image features in various brain regions and at different levels. The performance of the proposed AD prediction method was tested on 131 patients with MCI with MRI scans taken at different time points. Overall, the findings show that the proposed method with longitudinal data and the hierarchical classification framework generate promising results for AD prediction.

## Materials

Data were downloaded from the Alzheimer’s disease Neuroimaging Initiative (ADNI) database (www.loni.ucla.edu/ADNI, PI Michael M. Weiner). ADNI was launched in 2003 by the National Institute on Aging (NIA), the National Institute of Biomedical Imaging and Bioengineering (NIBIB), the Food and Drug Administration (FDA), private pharmaceutical companies and non-profit organizations, as a $60 million, 5-year public–private partnership. The primary goal of ADNI has been to test whether serial MRI, PET and other biological markers are useful in clinical trials of MCI and early AD. Determination of sensitive and specific markers of very early AD progression is intended to aid researchers and clinicians to develop new treatments and monitor their effectiveness, as well as lessen the time and cost of clinical trials. ADNI subjects aged 55 to 90 from over 50 sites across the US and Canada participated in the research and more detailed information is available at www.adni-info.org.

T1-weighted MRI images were used in this study. The scanning parameters for the 1.5T MRI images can be found in ref. 31. A total of 131 subjects with MCI (70 MCIc and 61 MCInc) from ADNI1 were considered in this study. Demographic information of the studied subjects is presented in Table 1. The diagnosis of the 61 MCInc subjects was MCI at all available time points (0–48 months). Moreover, the diagnosis of the 70 MCIc subjects was MCI at baseline but conversion to AD was reported after baseline within 6, 12, 24, 36, or 48 months, and without reversion to MCI or NC at any available follow-up (0–48 months). From the 70 MCIc subjects, 11 subjects were converted to AD within the first 6 months, 28 subjects were converted to AD between the 6 and 12 months follow-up, 17 subjects were converted to AD between the 12 and 18 months follow-up, 7 subjects were converted to AD between the 18 and 24 months follow-up, and the remaining 7 subjects were converted to AD between the 24 and 36 months follow-up. The number of MCI subjects who converted from MCI to AD during different time points is displayed in Table 2. The MRI scans at the baseline visit, 6, 12, 24, 36, and 48 months were used in the AD prediction when available. A longitudinal study usually covers a relatively long period of time in the field of health sciences; some individuals almost always miss their scheduled visits or date of observation. Therefore, the sequence of observation times may vary across individuals^19^. The details of subject number at different time points are listed in Table 3.

## Methods

### Preprocessing

Given that the intensity values in MRI images do not indicate a fixed meaning and widely vary within or between subjects, the MRI images were preprocessed with the following steps. First, the N3 method^32^ was applied to remove bias field artifacts from the images. Second, a two-step method^33^ was used to normalize the intensity values. Third, all images were spatially normalized to the publicly available ICBM152 average^34^ via FLIRT (http://www.fmrib.ox.ac.uk/fsl/). The images were subsequently aligned to a standard template space with an image size of 193 × 229 × 193 and a voxel size of 1 mm × 1 mm × 1 mm. Subsequently, the non-brain tissues were removed with the skull stripping method proposed in ref. 35. The intensity values were normalized again by the following steps. The intensity values for the brain region were calculated at 0.1% and 99.9% quantiles; both values were used to linearly scale the intensity values of voxels to the range of [0, 100]. Finally, for the simplicity of the proposed method, MRI data obtained before the missing time points were used as the missing data. For instance, if MRI scans are obtained at baseline visit, 6, 24, and 36 months, then we used the MRI scans from the 6 and 36 months as data for the 12 and 48 months, respectively. Thus, the number of time points remained the same among all the subjects considered for data collection.

### Basic idea of LMHC

In this study, voxel intensities in MRI images were used as features for classification. The voxel intensity is high dimensional, which consists of much more voxels (193 × 229 × 193 ≈ 8.53 × 10^6^) than the subjects (that is, hundreds at most). If all voxel intensities are used as classification features, the high dimensionality features will likely degrade the classification capability of the classifier. To solve this problem, a common strategy used is to select a set of useful voxels and apply a supervised classifier on these voxels to perform classification. However, an optimal subset of discriminative features is difficult to find by using only a single global classifier given that the discriminative features from the high dimensional neuroimaging data may lie in multiple low-dimensional feature subspaces^2^. Moreover, disease-induced structural changes may occur at some relatively large regions of the brain^36^,^37^. Therefore, the spatial information found in several voxel-grouped local regions should be considered to enhance the classification accuracy.

To address the abovementioned problems, we proposed a longitudinal measurement-based hierarchical classification framework that hierarchically combined multiple individual classifiers for more accurate AD prediction. Specifically, the logical regression classifier (LRC), which is easily used and trained, was employed to design each individual classifier. To select a set of informative voxels, the longitudinal data from different time points (MRI data of MCI subjects after the conversion were also included) were used to train each individual classifier. With the selected voxel set, a hierarchical classification framework can be built on the selected voxel sites with the longitudinal data at time points that are at least 6 months ahead of the conversion. Given that each voxel or region feature defines a subspace of the whole brain feature space, each individual classifier can be trained more easily in a much smaller subspace, and thereby substantially improving in the dimensionality-to-subject ratio. The accuracy of the final classification can be further improved by replacing a single classifier with a hierarchical classification framework.

**Figure 2:**
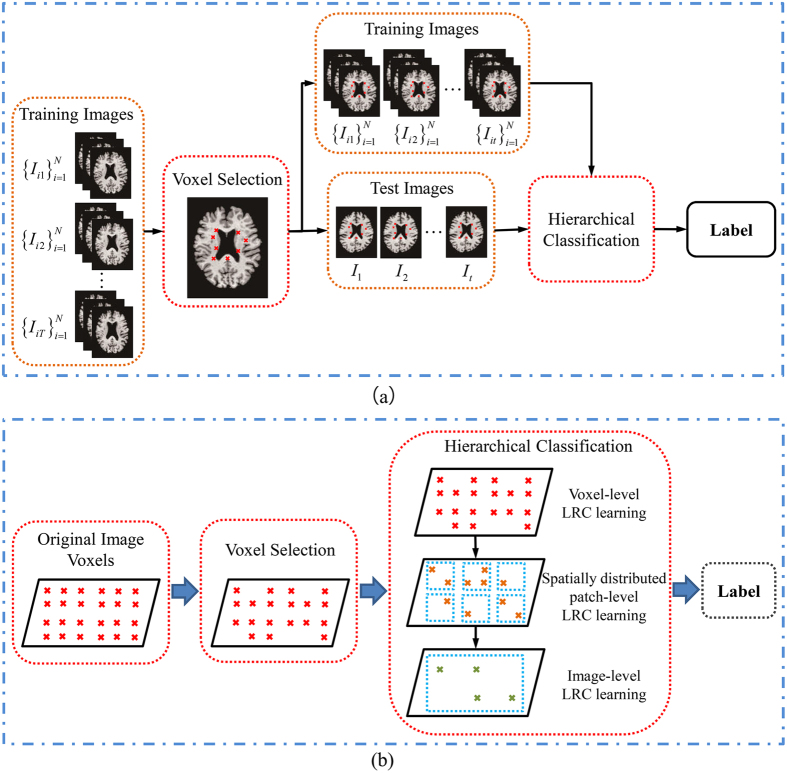
(**a**) Flowchart and (**b**) illustration of the proposed LMHC method.

The overall schematic of the proposed classification method is shown in Fig. 2. The method consists of two fundamental steps: a voxel selection step that selects a good subset of MRI voxels for AD conversion prediction and a classification step that uses a hierarchical framework to make the final prediction. We provide details for each step in the following sections.

### Selection of significant voxels

For accurate classification, the most useful voxels among all the MRI voxels were selected, whereas the noisy ones were excluded. For voxel selection, we used LRC on the longitudinal data. Given a training image set, *X* = {*I*_*ij*_, *L*_*i*_}_*i*=1, …, *N, j*=1, …, *T*_, for the longitudinal data, the longitudinal feature of a voxel site *v* of the images is represented as 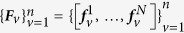, where *N* is the subject number, *T* is the number of time points, *L*_*i*_ ∈ {0, 1} is the label of the *i*th image, *n* is the voxel number, and 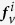 is a feature vector containing *T* elements. In this voxel selection step, the longitudinal data from different time points were included because the data from MCI to AD status can provide important conversion information on the classification of MCIc and MCInc. In addition, the longitudinal feature of each voxel site in the training images was used to learn an LRC, and the longitudinal features in the training images were fed to their corresponding classifiers to obtain a confident value for each voxel site. The confident values ranged from 0 to 1. Finally, the voxel sites with confident values higher than the threshold *t*_*s*_ were selected. Specifically, the cost function of LRC on the longitudinal features at voxel site *v* was calculated as


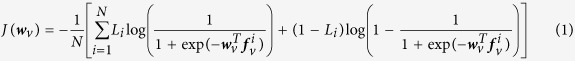


Given that no closed-form technique can be used to solve for the minimum of *J*(***w***_*v*_), we used the gradient descent method to iteratively optimize eq. (1). For the training image set, *X* = {*I*_*ij*_, *L*_*t*_}_*i*=1, …, *N, j*=1, …, *T*_, and its corresponding longitudinal feature set, 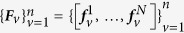, the classification result of LRC for subject *i* at voxel site *v* was defined as


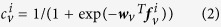


Thus, the confident value at voxel site *v* was calculated as


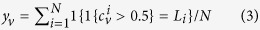


Finally, the significant voxels were selected as *V*_*s*_ = {*v*|*y*_*v*_ > *t*_*s*_}.

### Hierarchical classification method

After selecting a set of significant voxels, a hierarchical classification framework was constructed on the selected voxel sites of the longitudinal data. In this step, only the longitudinal data at time points that are at least 6 months ahead of the conversion were used because of their importance in predicting the conversion of MCI in the clinic. In the hierarchical classification framework, a three-level classifier was built for forming decisions: voxel, patch, and image levels. For the first-level classifier, a classifier was built for each longitudinal feature at every significant voxel site. For the second- and third-level classifiers, the outputs from the lower-level classifiers were fed to the corresponding upper-level classifier (Fig. 2(b)). Specifically, for the voxel-level classification, an LRC was independently trained for each significant voxel site with the longitudinal feature as input. The output coming from the voxel-level LRC is a confident value, which was obtained as the output in the voxel selection step. We selected the confident values higher than the threshold *t*_*h*_ as inputs for the patch-level classifier. To incorporate the spatial information, an image with the original image size (193 × 229 × 193) was generated. In this image, the values at the selected voxel sites (that is, voxel sites with confident values higher than *t*_*h*_) were set according to the corresponding confident values, whereas the rest of the values were set to 0. Subsequently, the confident values inside a patch with size *w* (image values equal to 0 were excluded) on the image were fed to an LRC for patch-level classification. Finally, the confident value from a patch-level classifier with a value higher than *t*_*h*_ was isolated and fed to the image-level classifier. The output obtained from the image-level classifier was the final decision for AD prediction. Notably, the threshold *t*_*h*_ at different levels shared the same value. Moreover, voxels that were not useful (i.e., *p* < *t*_*h*_) for the classification were discarded, and each patch classifier covered various regions of the different brain areas.

### Summary of LMHC

To elucidate the concept of LMHC, we provided a pseudo-code for LMHC, as illustrated in Algorithm 1.

**Algorithm 1**. LMHC


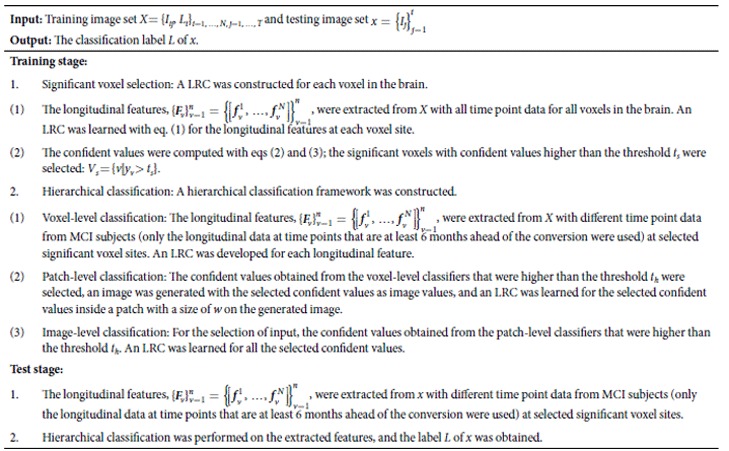


## Results

### Experimental setting

The proposed method was evaluated with two nested cross-validation loops (10-fold for each loop)^9^. Specifically, for the external 10-fold cross-validation, all subject samples were divided into 10 subsets with the same proportion of each class label. For each run, all samples within one subset were successively chosen as the testing set, whereas the remaining samples in the other nine subsets were combined and used as the training set for voxel selection and classification. The final classification results were reported as the mean results from each run. Moreover, parameter tuning was evaluated with the inner 10-fold cross-validation on the training set. In particular, the training set can be further split into a training part and a validation part for each run of the external 10-fold cross-validation. By varying the values of the different parameters, the proposed classifier was developed using the samples in the training part. The classification results were obtained during validation. The parameters with the maximum average classification accuracy during validation were selected. Notably, all longitudinal data (MRI data of MCI subjects after the conversion were also included) on a subject were used in the training step to select a set of informative voxels. However, given that only NC and MCI data were available in practice for AD prediction, the longitudinal data at time points that were at least 6 months ahead of the conversion were used to train hierarchical classifiers. In addition, the longitudinal data at time points that were at least 6 months ahead of the conversion were used in the validation and testing steps.

In the experiments, four measurement criteria were applied to evaluate the classification performance: sensitivity (SEN), specificity (SPE), accuracy (ACC), and the area under the receiver operating characteristic (ROC) curve (AUC). Specifically, the accuracy is the proportion of subjects correctly predicted among all the studied subjects. The sensitivity is the proportion of correctly predicted MCIc, whereas the specificity is the proportion of correctly predicted MCInc.

### Parameter optimization

The parameter settings of the proposed method were carefully considered to achieve optimum performance in our experiments. A summary of the parameter settings in the proposed method is presented in Table 4.

In the step for significant voxel selection, the threshold *t*_*s*_ was selected from the group {0.5, 0.55, 0.6, 0.65} to determine a set of significant voxels. Generally, the number of significant voxels is reduced when the threshold *t*_*s*_ is increased. However, few significant voxels were left when *t*_*s*_ > 0.7 was considered in our experiment. Therefore, the maximum value of *t*_*s*_ selected was 0.65 to reserve the useful information for the following classification step. Additionally, the threshold *t*_*h*_ and patch size *w* were set to 0.5 and 5, respectively. Table 5 shows the classification results with different *t*_*s*_ values. This table also shows that ACC is improved by the increase in *t*_*s*_. In addition, all classification measurements reached their highest values when *t*_*s*_ = 0.65. Thus, the threshold *t*_*s*_ was fixed at 0.65 in the subsequent experiments to reduce the feature dimensionality and reserve useful information for the following classification step.

In the hierarchical classification step, the threshold *t*_*h*_ and patch size *w* are two crucial parameters that should be carefully determined. Thus, the two experiments were conducted to optimize the threshold *t*_*h*_ and patch size *w*, separately, during classification. In the first experiment, the proposed method was tested with different *t*_*h*_ values from 0 to 0.65. Moreover, the threshold *t*_*s*_ and patch size *w* were set to 0.65 and 5, respectively. Table 6 shows that the classification results with the threshold *t*_*h*_ = 0.5 were higher than the classification results without the threshold (*t*_*h*_ = 0). However, the classification accuracy was reduced when the threshold *t*_*h*_ increased at *t*_*h*_ > 0.5. A threshold of *t*_*h*_ = 0.5 was chosen for the subsequent experiments. In the second experiment, the patch size *w* was varied from 1 to 3, 5, 7, 9, and 11 to test the classification performance with these different values. In addition, the thresholds *t*_*s*_ and *t*_*h*_ were set to 0.65 and 0.5, respectively. As shown in Table 7, the ACC was improved by increasing *w* from 0 to 5, but further increasing the patch size to 7, 9, and 11 reduced ACC. Therefore, the patch size *w* was fixed at 5 for the subsequent experiments.

### Effectiveness of the use of longitudinal data

**Figure 3:**
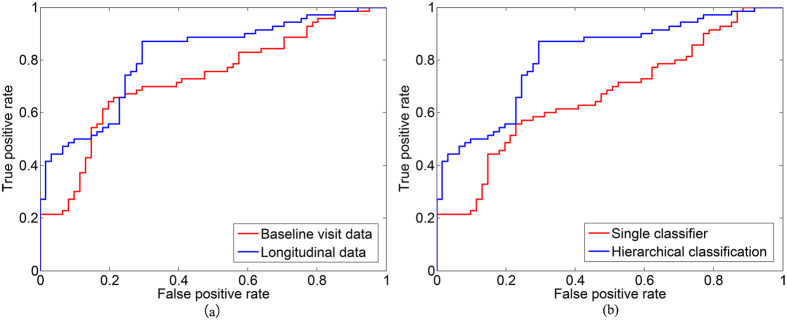
ROC curves for the classification of MCIc and MCInc obtained with (**a**) baseline visit data and longitudinal data and (**b**) a single classifier and hierarchical classification.

To assess the effectiveness of the use of longitudinal data, the classification performance was evaluated with MRI scans of MCI subjects from the baseline visit data and the longitudinal data. In the experiment for baseline visit data, only the baseline visit data were used for comparison in the significant voxel selection and hierarchical classification steps. For fair comparison, we also used two nested cross-validation loops (10-fold for each loop) to carefully select the parameters with optimal performance for the baseline visit data. Moreover, the parameters *t*_*s*_, *t*_*h*_, and *w* were set to 0.65, 0.5, and 5, respectively, in accordance with the proposed method by using longitudinal data. The classification results of the proposed method with the baseline visit data and longitudinal data are shown in Table 8 and Fig. 3(a). The proposed method with longitudinal data consistently outperforms the proposed method that used baseline data in terms of ACC, SEN, SPE, and AUC. High SEN values indicated high confidence in AD prediction, which will significantly benefit the application of the method in real-life situations. The proposed method with longitudinal data significantly improved the sensitivity value (nearly 17% higher than the proposed method with baseline data). This high sensitivity may be advantageous for confident AD prediction and useful in practical applications.

### Effectiveness of the hierarchical classification framework

To evaluate the effect of the hierarchical classification framework on the classification performance, we compared the obtained classification results by building a single global classifier and a hierarchical classification framework. For fair comparison, both classification methods were used on the same significant voxel set. We then used an LRC and the proposed hierarchical classification framework, respectively, to achieve the final classification result. Moreover, the parameters *t*_*s*_, *t*_*h*_, and *w* were set to 0.65, 0.5, and 5, respectively, in the proposed hierarchical classification method. Table 9 shows the classification results with respect to the single global classifier and the hierarchical classification framework. In addition, the ROC curves of different methods for classification of MCInc versus MCIc are illustrated in Fig. 3(b). These results demonstrate that the hierarchical classification framework performs better than the single classifier.

### Computation cost

In this study, the experiments were implemented on a standard PC with an Intel Xeon E5-2620 v3 processor at 2.40 GHz. To classify a subject in the testing step, the processing time was approximately 5 min; of which, 4 min was allotted for intensity and spatial normalization, and 1 min was allotted for the actual classification. In the training step, 6 threads were used to perform voxel selection and hierarchical classifier learning, the parameters *t*_*s*_, *t*_*h*_, and *w* were set to 0.65, 0.5, and 5, respectively. The total processing time for 118 MCI subjects was approximately 10 h; of which, 8 h was allotted for voxel selection, and 2 h was allotted for hierarchical classifier learning.

## Discussion

We proposed a novel classification method for AD prediction. Our study is twofold. First, we investigated the longitudinal images of 131 individuals with MCI to obtain important information on the longitudinal change. The data were subsequently used to classify MCI subjects into either MCIc or MCInc. The longitudinal change is a crucial factor in the prediction of possible conversion from MCI to AD. This factor is widely used in AD conversion analysis^20^,^23^,^38^. In most methods, the selected time points of MRI scans must be the same among individuals to capture changes in the longitudinal data. However, given that a longitudinal study in the field of health sciences commonly covers a relatively long period of time, some individuals may miss their scheduled visits^19^. Therefore, the same time points of MRI scans are difficult to implement across individuals. In the present study, the voxel intensities in the MRI images from the longitudinal data were used as features. The MRI data gathered before the missing time point were used as the missing data. Thus, we could use the longitudinal data at different time points. Second, we developed a hierarchical classification framework to address the high feature dimensionality issue and incorporate spatial information, thereby improving the classification accuracy. Unlike other hierarchical classification methods^16^,^30^, our proposed strategy established multiple and multilevel classifiers with supervised learning and suitable thresholds to address the issues of high feature dimensionality and sensitivity to small changes. These characteristics enhance the classification accuracy. In the experiment, the classification results with a threshold *t*_*h*_ are consistently higher than the classification results without this threshold (Table 6). Therefore, unusable information can be discarded through the proposed hierarchical classification method with a suitable threshold.

In the hierarchical classification framework, the patch size *w* was adjusted to optimize the classification performance. Small patches may lack the required information for good performance in patch-level classification, and numerous patches or patch-level classifiers will significantly increase the computational cost for the classification. More redundant or even confounding information may be included in a large patch, thereby affecting the localization of informative brain regions and the ensemble classification results. In our experiment, the classification results with *w* = 5 were higher than those of other patch sizes (Table 7). Therefore, a moderate-sized patch was optimum in the proposed method as compared with other patch sizes.

Comparisons of baseline visit data versus longitudinal data and single classifier versus hierarchical classification were conducted to evaluate the performance of the proposed method in this study. Table 8 and Fig. 3(a) show that the classification results from longitudinal data are higher than those obtained with baseline visit data. These findings suggest that longitudinal change is a crucial factor for the prediction of future conversion of MCI to AD. Moreover, our experimental results show that the method with the hierarchical classification framework performs better than a single global classifier, probably because the hierarchical classification framework can better utilize local features and classifier decisions. In the hierarchical classification framework, the local spatial contiguity of image features is important during classification with a hierarchical spatial structure built from voxels to larger brain regions. The hierarchical spatial structure can utilize the local information better than the ROI-based methods^30^. An ensemble method can also improve the generalizability and robustness of individual classifiers for better classification decisions as compared with individual classifiers^2^. Therefore, the proposed hierarchical ensemble method can utilize the local features and make better classifier decisions than a single global classifier.

In the training stage, the proposed method requires that a classifier is trained for each voxel in the brain to select significant voxels and construct a hierarchical classification framework to train a hierarchical classifier. These two steps are off-line procedures; thus, each step is performed only once but used for all testing images. Moreover, both steps were run in multi-threads in this study, thereby significantly decreasing processing time.

Table 10 shows that the classification results of the proposed method (ACC = 79.4%, SEN = 86.5%) are comparable to the results of recently published papers. Tang *et al*.^39^ and Wee *et al*.^40^ extracted vertex-based features from MRI scans obtained from baseline visit data to classify MCI subjects into either MCIc or MCInc, and an accuracy of 75.0% and 75.1% were obtained, respectively. Liu *et al*.^30^ proposed a hierarchical ensemble classification method to combine multilevel classifiers through gradual integration of a large number of features from local brain and interbrain regions. MRI scans from baseline visit data were used for AD prediction, and an accuracy of 64.8% was attained. Suk *et al*.^16^ first used the deep learning method to learn a high-level latent and shared feature representation. They then constructed a hierarchical classifier for the classification of MCIc versus MCInc. MRI and PET markers were included, and an accuracy of 75.9% was obtained. Zhang *et al*.^3^ used longitudinal data to predict future conversion of patients with MCI, and an accuracy of 78.4% was obtained. Recently, Korolev *et al*.^41^ incorporated risk factors, cognitive and functional assessments, MRI, and plasma proteomic data for AD prediction. They obtained a high accuracy of 80.0%. Although the accuracy of our study is less than that of Korolev *et al*.’s study, our study is comparable to their model that incorporated only MRI data (ACC = 71.4%). Therefore, incorporating many data sources can potentially improve our prediction model in the future. However, only the results of different methods in literature are listed. Direct comparison of the performances of different methods is not reasonable because various datasets and methods for extracting features and building classifiers were used. Nonetheless, the proposed method showed the highest sensitivity and the second highest accuracy among the methods for MCInc/MCIc classification. These observations implied that the proposed method can potentially enhance confidence in AD prediction.

**Figure 4:**
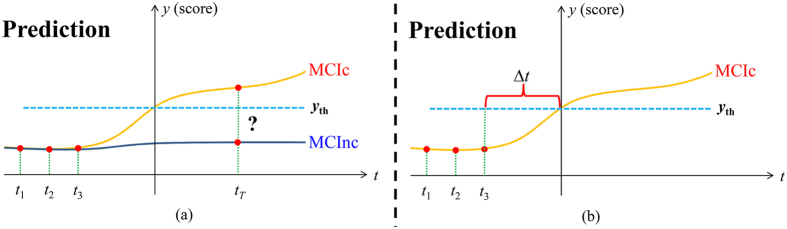
An example using longitudinal data to predict (**a**) AD conversion and clinical scores; (**b**) a rough time of AD conversion.

In this research, only MRI data were used. The data can be expanded to include other image modality data in future studies. Different image modalities can provide complementary information for disease diagnosis. Moreover, other data sources, such as clinical scores, genetic data, and demographic data, can be included to improve our prediction model in the future. Further advanced classifier ensemble methods, such as sparse multiple kernel learning^42^, can be investigated in future work to improve the classification performance. Finally, our method exclusively focused on the MCIc/MCInc classification. In the future, we aim to incorporate clinical scores, such as the Alzheimer’s Disease Assessment Scale-Cognitive subscale and the Mini-Mental State Examination, to construct a joint regression and classification model. For instance, we can simultaneously perform AD and clinical score prediction with such a model. Furthermore, we can estimate the time when an MCIc subject develops AD by using the longitudinal data and the constructed model (Δ*t* shown in Fig. 4).

## Conclusion

This study presented a novel AD prediction method based on LMHC. Longitudinal images from individuals with MCI were investigated to obtain important information on the longitudinal changes for classifying MCI subjects into MCIc and MCInc. A hierarchical framework was introduced into the classifier to address the high feature dimensionality and incorporate spatial information for improved prediction accuracy. The performance of the proposed AD prediction method was tested on 131 patients with MCI with MRI scans at different time points. Our experimental results showed that longitudinal data and the hierarchical classification framework of our proposed method can improve the classification performance. To our knowledge, previous studies have not combined these two characteristics for AD prediction.

## Additional Information

**How to cite this article**: Huang, M. *et al*. Longitudinal measurement and hierarchical classification framework for the prediction of Alzheimer’s disease. *Sci. Rep.* **7**, 39880; doi: 10.1038/srep39880 (2017).

**Publisher's note:** Springer Nature remains neutral with regard to jurisdictional claims in published maps and institutional affiliations.

## Figures and Tables

**Figure 1:**
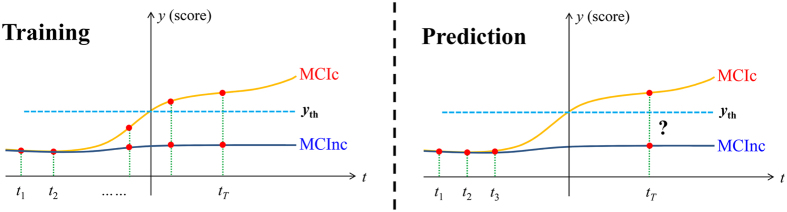
An example using longitudinal data to predict AD conversion.

**Table 1:** Demographic information of the studied subjects from the ADNI database.

Diagnosis	Number	Age	Gender (M/F)	MMSE
MCIc	70	74.26 ± 7.55	42/28	26.46 ± 1.76
MCInc	61	75.85 ± 6.49	32/29	27.05 ± 1.78

**Table 2:** Number of MCI subjects who developed to AD during different time points (6 m, 12 m, 18 m, 24 m, and 36 m represent 6, 12, 24, and 36 months, respectively).

Time point	First 6 m	6–12 m	12–18 m	18–24 m	24–36 m	Total
number	11	28	17	7	7	70

**Table 3:** Number of MCIc and MCInc subjects at different time points (6 m, 12 m, 18 m, 24 m, 36 m, and 48 m represent 6, 12, 24, 36, and 48 months, respectively).

	Baseline	6 m	12 m	18 m	24 m	36 m	48 m	Total
MCIc	70	61	65	52	52	31	8	339
MCInc	61	55	49	30	27	12	1	235
Total	131	116	114	82	79	43	9	574

**Table 4:** Summary of the parameter settings in the proposed method for AD prediction.

Parameter	Description	Setting
*t*_*s*_	Threshold in significant voxel selection	0.65
*t*_*h*_	Threshold in hierarchical classification framework	0.5
*w*	Patch size (*w* × *w* × *w*)	5

**Table 5:** Classification results of the proposed method with different *t*_*s*_ values.

*t*_*s*_	0.5	0.55	0.6	0.65
ACC (%)	55.1	56.3	62.1	**79.3**
SEN (%)	62.0	67.6	64.2	**87.7**
SPE (%)	55.9	45.3	51.4	**73.1**

**Table 6:** Classification results of the proposed method with different *t*_*h*_ values.

*t*_*h*_	0	0.5	0.55	0.6	0.65
ACC (%)	73.8	**79.3**	74.6	78.0	76.5
SEN (%)	83.8	**87.7**	85.0	84.1	83.8
SPE (%)	62.3	**73.1**	62.7	71.1	68.7

**Table 7:** Classification results of the proposed method with different *w* values.

*w*	1	3	5	7	9	11
ACC (%)	74.0	74.0	**79.3**	76.3	76.3	74.8
SEN (%)	86.8	79.7	87.7	86.2	90.2	**92.8**
SPE (%)	65.1	69.2	**73.1**	69.0	63.2	49.3

**Table 8:** Classification results of the proposed method with baseline visit data and longitudinal data.

Method	ACC (%)	SEN (%)	SPE (%)	AUC
Baseline visit	71.7	69.9	77.7	0.754
Longitudinal data	79.4	86.5	78.2	0.812

**Table 9:** Comparison of single classifier and hierarchical classification for MCInc versus MCIc classification.

Method	ACC (%)	SEN (%)	SPE (%)	AUC
Single classifier	64.9	54.9	78.0	0.712
Hierarchical classification	79.4	86.5	78.2	0.812

**Table 10:** Comparison of MCInc/MCIc classification accuracy in literature.

Method	Subjects (MCInc/MCIc)	Data source	Features	Classifier	ACC (%)	SEN (%)	SPE (%)
Korolev *et al*.^41^	120/139 (baseline visit)	Risk factors, cognitive and functional assessments, MRI, plasma proteomic data	ROI-wise	Probabilistic multiple kernel learning	**80.0**	83.0	76.0
Tang *et al*.^39^	87/135 (baseline visit)	MRI	Vertex-based	LDA	75.0	77.0	71.0
Liu *et al*.^30^	128/ 76 (baseline visit)	MRI	Voxel-wise	Hierarchical ensemble	64.8	22.2	89.6
Suk *et al*.^16^	128/76 (baseline visit)	MRI, PET	Voxel-wise	Hierarchical ensemble	75.9	48.0	**95.2**
Wee *et al*.^40^	111/89 (baseline visit)	MRI	Vertex-based	SVM	75.1	63.5	84.4
Zhang *et al*.^3^	50/35 (longitudinal data)	MRI, PET, cognitive scores	ROI-wise	SVM	78.4	79.0	78.0
Proposed method	61/70 (longitudinal data)	MRI	Voxel-wise	Hierarchical ensemble	79.4	**86.5**	78.2
